# Impact of type 2 diabetes mellitus on acute levodopa responsiveness in Parkinson's disease: A Multicenter retrospective study

**DOI:** 10.1016/j.prdoa.2026.100502

**Published:** 2026-09-15

**Authors:** Olga Stockmann, Lan Ye, Carsten Buhmann, Fabian Maass, Christoph van Riesen, Christiane Weinrich, Florian Wegner, Matthias Höllerhage, Monika Pötter-Nerger, Martin Klietz

**Affiliations:** a Department of Neurology, Hannover Medical School, Carl-Neuberg-Straße 1, 30625 Hannover, Germany; b Department of Neurology, University Medical Center Hamburg-Eppendorf, 20246 Hamburg, Germany; c Department of Neurology, University Medical Center Göttingen, 37075 Göttingen, Germany

**Keywords:** Parkinson's disease, Type 2 diabetes mellitus, Levodopa challenge, Motor response

## Abstract

**Background:**

Type 2 diabetes mellitus (T2DM) is a common comorbidity in Parkinson's disease (PD) and may influence the progression and dopaminergic function, suggesting a potential impact on levodopa responsiveness.

**Objective:**

To examine the impact of T2DM status on levodopa responsiveness in an acute levodopa challenge in PD.

**Methods:**

We retrospectively analyzed the total and subdomain motor responses in the acute levodopa challenge in 136 PD patients with normal glycemic status, prediabetes, or T2DM across three German centers.

**Results:**

Levodopa responsiveness did not differ significantly among the three glycemic groups. The only between-group difference was more severe rest tremor in T2DM patients in the OFF state, which was no longer present in the ON state due to greater levodopa-induced improvement.

**Conclusion:**

An acute levodopa challenge remained similarly effective across glycemic groups in PD, suggesting that T2DM does not substantially modify acute dopaminergic treatment response. Future studies should also evaluate chronic response to levodopa across different glycemic statuses.

## Highlights

• Although type 2 diabetes mellitus (T2DM) is a highly prevalent comorbidity in Parkinson's disease (PD), its impact on dopaminergic responsiveness in PD remains poorly understood. This study aimed to investigate the influence of T2DM status on levodopa responsiveness in patients with PD.
• A retrospective multicenter study was conducted across three German movement disorders centers. Data from 136 well-characterized PD patients were analyzed, stratified by glycemic status: normal glycemia, prediabetes, and T2DM, based on results from acute levodopa challenge tests.
• Our findings show that levodopa responsiveness is preserved across all glycemic groups, with no significant differences in overall motor improvement or in most motor subdomains. These results suggest that T2DM does not substantially alter the acute dopaminergic response to levodopa in patients with PD.
• T2DM does not alter acute levodopa response in Patients with PD.

## 1 Introduction

Parkinson's disease (PD) is the second most common neurodegenerative disorder [1] and is often accompanied by numerous comorbidities, frequently including type 2 diabetes mellitus (T2DM) [2]. Accumulating evidence indicates that T2DM is also a potential risk factor for PD. Beyond its clinical PD-association with faster motor progression and greater cognitive impairment, T2DM has also been biologically linked to dopaminergic denervation and hyperglycemia-related postsynaptic alterations, raising the possibility that T2DM may influence levodopa responsiveness in PD [3], [4], [5], [6]. A previous study has also shown that PD patients with T2DM required higher levodopa treatment doses than the ones without T2DM [7]. Given that Levodopa is the gold standard and first-line pharmacologic therapy for PD [8], plays a key role in diagnosis, particularly in the differential diagnosis of atypical parkinsonism [9], and guides decisions regarding invasive approaches such as deep brain stimulation [10], it is important to clarify the impact of T2DM on levodopa responsiveness. In the present study, we systematically examined an acute levodopa challenge from the total motor response and its subdomains to the glycemic status of the patients.

## 2 Methods

A retrospective multicentric study with 136 PD patients was conducted to evaluate an acute levodopa challenge across three German centers (Hannover, Hamburg and Göttingen). These PD patients were stratified into three groups based on glycemic status according to American Diabetes Association criteria [11]: normal glycemic status (HbA1c <5,7%) (*n* = 76), prediabetes (PreDM) (HbA1c 5,7-6,4%) (*n* = 21), and T2DM (patients with diagnosed type 2 diabetes mellitus) (*n* = 39). PD was diagnosed by a movement specialist according to Movement Disorders Society (MDS) diagnostic criteria [12]. The acute levodopa challenge was performed using 200/50 mg immediate-release levodopa/benserazide (Madopar LT®) according to a standardized protocol [9]. Levodopa response was defined as the percentage improvement in MDS-Unified Parkinson's Disease Rating Scale Part III (MDS-UPDRS III) scores from OFF to ON, with <30% classified as negative (non-response) and ≥ 30% as positive response (good response). In addition to the total score, MDS-UPDRS III subdomains (speech, rigidity, rest tremor, other tremors, gait and posture, bradykinesia) were examined.

Demographic and clinical data included age at onset, age at presentation, sex, disease duration, Montreal Cognitive Assessment (MoCA), Hoehn and Yahr stage, and MDS-UPDRS III scores in OFF and ON states. Antidiabetic medication was recorded for patients with T2DM.

The study was approved by the local ethics committee (Hannover No. 866_Bo_K_2019) and conducted in accordance with the Declaration of Helsinki. All participants gave their written informed consent.

### 2.1 Statistical analysis

All analyses were performed using JASP (version 0.19.3) and R Studio (version 2024.12.1). Normality was assessed with the Shapiro–Wilk test. Group comparisons were conducted using one-way ANOVA for normally distributed variables or the Kruskal–Wallis test for non-normally distributed data. For significant one-way ANOVA results, post-hoc pairwise comparisons were performed with Bonferroni correction. For significant Kruskal–Wallis tests, Dunn's post-hoc test with Bonferroni correction was performed for pairwise comparisons. Categorical variables were analyzed using Chi-Square tests with post-hoc pairwise comparisons if overall significance was observed. One-way analysis of covariance (ANCOVA) with Bonferroni correction whenever adjustment for covariates was required.

## 3 Results

As presented in Table 1, baseline demographic and clinical characteristics were largely comparable among the groups, except for a shorter disease duration in patients with prediabetes compared to those with T2DM (PD + PreDM 4.7 ± 6.6 years vs. PD + T2DM 7.9 ± 4.8 years, Mann-Whitney *U* test *p < 0.05).* HbA1c differed as expected across groups: PD + NG 5.2 ± 0.3%, PD + PreDM 5.9 ± 0.2%, and PD + T2DM 6.9 ± 1.2%; all pairwise comparisons were significant (post-hoc tests, *p < 0.05*).

**Table 1:** Demographic and clinical characteristics of the participants.

	PD + NG (*N* = 76)	PD + PreDM (*N* = 21)	PD + T2DM (*N* = 39)	*P* value
Age at onset (years)	59.5 ± 12.2	61.2 ± 12.0	57.3 ± 10.6	0.23
Age at presentation (years)	66.0 ± 10.7	65.9 ± 10.9	65.1 ± 8.2	0.76
Sex (male %)	57.9	71.4	76.9	0.10
Duration of the disease (years)	6.1 ± 5.6	4.7 ± 6.6⁎	7.9 ± 4.8⁎	**0.01**
Hoehn-Yahr	2.6 ± 0.9	2.7 ± 1.0	2.5 ± 0.8	0.85
MDS-UPDRS III (on)	26.8 ± 15.0	27.9 ± 14.4	25.7 ± 13.3	0.93
MDS-UPDRS III (off)	38.1 ± 16.7	39.6 ± 16.2	39.2 ± 12.1	0.72
MoCA	24.5 ± 4.2	22.7 ± 5.2	25.2 ± 3.8	0.22
HbA1C (%)	5.2 ± 0.3⁎	5.9 ± 0.2⁎	6.9 ± 1.2⁎	**0.00**

**Abbreviations:** PD + NG: PD patients with normal glycemic status; PD + PreDM: PD patients with prediabetes; PD + T2DM: PD patients with type 2 diabetes mellitus. PD Parkinson's disease; T2DM type 2 diabetes mellitus. MDS-UPDRS Movement Disorders Unified Parkinson's disease Rating Scale; MoCA Montreal cognitive assessment.

⁎ indicate the groups with statistically significant differences in post hoc analysis.

All three glycemic groups showed a significant reduction in MDS-UPDRS III total scores and all the subdomains following the acute levodopa challenge. Noteworthy, when comparing across glycemic groups, patients with T2DM exhibited higher rest tremor scores than those with prediabetes in the OFF state (Kruskal-Wallis*, p < 0.05*) (Fig. 1A). No other between-group differences were detected in either the OFF (Fig. 1A) or ON state (Fig. 1B). After adjusting for disease duration, OFF-state scores remained comparable across all motor domains except for rest tremor. Patients with T2DM continued to exhibit higher baseline rest tremor scores than those with prediabetes (ANCOVA, *p < 0.01*) and those with normal glycemic status (ANCOVA, *p < 0.05*). After adjusting for the disease duration in the ON state there were no significant differences between the groups in either the total motor domain or any motor subdomain, consistent with the results from the unadjusted ANOVA.

**Fig. 1:**
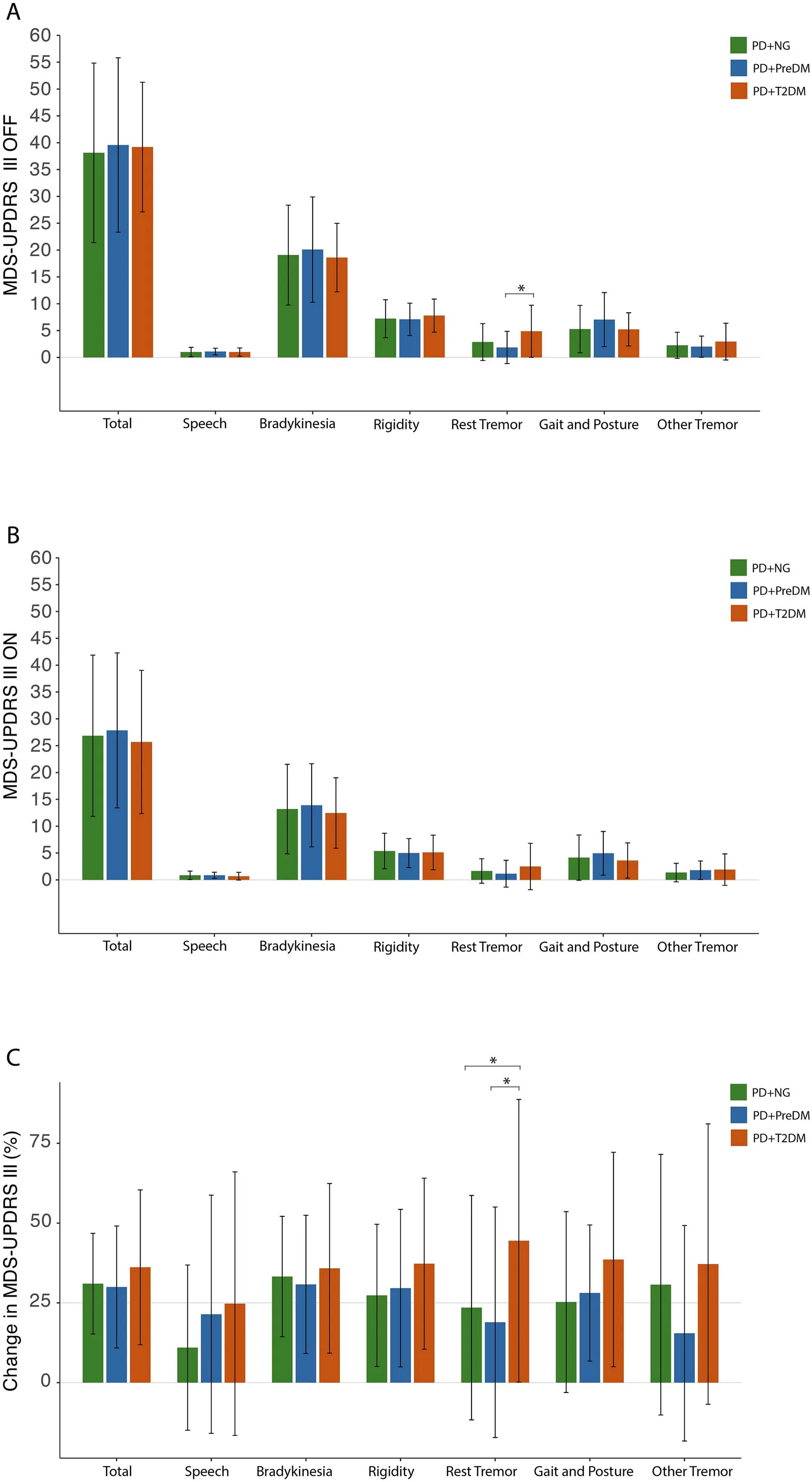
Change in MDS-UPDRS III scores between OFF and ON states during the acute levodopa challenge in PD patients with normal glycemic status, prediabetes, and T2DM. **(A)** Absolute MDS-UPDRS III scores (total and subdomains) in PD patients with normal glycemic status (PD + NG), prediabetes (PD + PreDM), and T2DM (PD + T2DM). Assessments were conducted in OFF during the acute levodopa challenge. Subdomains include Speech, Bradykinesia, Rigidity, Rest Tremor, Gait and Posture, and Other Tremor. **(B)** Absolute MDS-UPDRS III scores (total and subdomains) in PD patients with normal glycemic status (PD + NG), prediabetes (PD + PreDM), and T2DM (PD + T2DM). Assessments were conducted in ON during the acute levodopa challenge. Subdomains include Speech, Bradykinesia, Rigidity, Rest Tremor, Gait and Posture, and Other Tremor. **(C)** Percentage change of the MDS-UPDRS III scores (total and subdomains) following the acute levodopa challenge. * Indicate statistically significant post-hoc differences between glycemic groups (*p < 0.05*). **Abbreviations:** PD + NG: PD patients with normal glycemic status; PD + PreDM: PD patients with prediabetes; PD + T2DM: PD patients with T2DM.

In addition, percentage improvement in MDS-UPDRS III scores from OFF to ON was calculated and patients with T2DM demonstrated a greater relative improvement in rest tremor compared to those with prediabetes and normal glycemic status. Otherwise, no differences among the three glycemic groups were observed for the total score or any other subdomains (one-way ANOVA, *p < 0.05*) (Fig. 1C).

Consistent with these findings, the proportion of patients demonstrating a positive response to the acute levodopa challenge in the total score and across all subdomains differed significantly between the three glycemic groups (Chi-Square. *p < 0.05*) with respect to rest tremor. Post hoc analyses revealed no significant differences between individual group pairs (Supplementary Fig. S1). This represented the only significant between-group difference observed.

Up to 82% of the patients with T2DM were treated with Metformin (Biguanides). An overview of antidiabetic medication use is presented in Supplementary Table S1. A further analysis of the impact of antidiabetic medication on levodopa responsiveness was not possible.

No correlation between HbA1c levels and levodopa responsiveness was detected.

## 4 Discussion

This is the first study to systematically assess levodopa responsiveness across a gradient of glycemic statuses within a well-characterized multicenter cohort. Our findings indicate that glycemic status did not significantly affect overall acute levodopa responsiveness or most motor subdomains. The only difference was more severe rest tremor in T2DM patients in the OFF state, a difference that largely disappeared after levodopa administration due to greater levodopa-induced improvement.

The pronounced levodopa responsiveness of rest tremor in PD patients with T2DM was unexpected, since tremor responsiveness in PD is typically heterogeneous or even absent. Rest tremor in PD reflects both dopaminergic basal ganglia and non-dopaminergic cerebello–thalamo–cortical contributions, with levodopa responsiveness determined by the relative contribution of these systems [13]. One hypothesis is that, in PD patients with T2DM, enhanced nigrostriatal dysfunction might shift tremor pathogenesis toward a more basal ganglia–driven, dopaminergic phenotype, resulting in greater OFF-state severity but stronger levodopa response in the ON state. Such a pattern would be consistent with a previous observation that patients with dopamine-responsive tremor had greater disease severity [14].

A previous study found no differences in bradykinesia, rigidity, or tremor between PD patients with and without T2DM but reported greater gait and postural instability [3]. Although this and our study showed only limited effects of T2DM, the affected motor domains differed. This might be explained by differences in disease duration and the grouping strategies used in their study compared to the present study.

Given the central role of levodopa responsiveness in the acute levodopa challenge in PD diagnosis and in evaluating candidates for advanced therapies, these results suggest that T2DM or prediabetes is unlikely to interfere with these clinical assessments.

A limitation of this study is that prediabetes patients were untreated, whereas T2DM patients were on antidiabetic therapy, potentially influencing group differences. These treatment imbalances are difficult to avoid in clinical cohorts, and the possibility that antidiabetic medications influence levodopa responsiveness cannot be fully excluded. For example, metformin has been proposed to have neuroprotective and geroprotective properties, it is conceivable that metformin treatment may have partially attenuated the potential detrimental effects of T2DM on Parkinsonian symptoms [15], [16]. Longitudinal studies with long-term levodopa treatment, extended follow-up, and comprehensive metabolic characterization may help to clarify whether chronic metabolic dysfunction interacts with dopaminergic pathways in ways not detectable in an acute levodopa challenge. The other limitation of this study is that, although differences in disease duration between patients with prediabetes and T2DM were accounted for using ANCOVA, a residual influence of disease duration cannot be completely ruled out. Matching the groups for disease duration might have further improved their comparability and, consequently, strengthened the validity of the findings.

In summary, our findings indicate that acute levodopa responsiveness in PD is preserved across glycemic status categories, including prediabetes and T2DM.

The following are the supplementary data related to this article.

**Supplementary Fig. S1:**
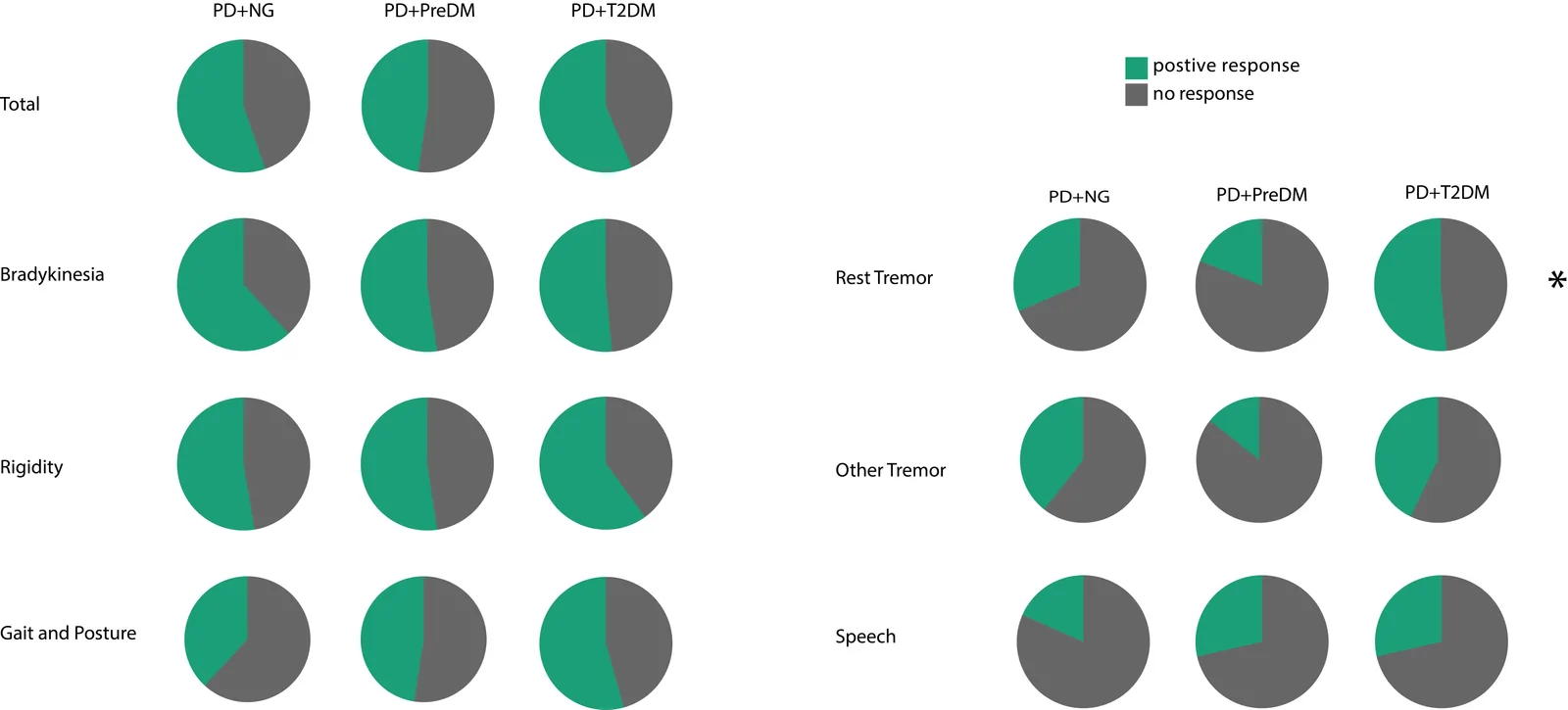
Proportion of patients with positive responses in the acute levodopa challenge across motor symptom categories in PD patients with and without diabetes. Pie charts illustrate the distribution of positive (green) versus no (gray) responses for the total as well as subdomains in PD patients with normal glycemic status (PD + NG), prediabetes (PD + PreDM), and T2DM (PD + T2DM). * Indicate overall group differences (*p < 0.05*). **Abbreviations:** PD + NG: PD patients with normal glycemic status; PD + PreDM: PD patients with prediabetes; PD + T2DM: PD patients with PD Parkinson's disease; T2DM type 2 diabetes mellitus.

Supplementary Material 2 Supplementary Table S1.

## Financial Disclosure/Conflict of Interest concerning the research related to the manuscript

The authors declare no financial disclosure and no conflicts of interest relevant to this work.

## CRediT authorship contribution statement

**Olga Stockmann:** Writing – original draft, Formal analysis, Data curation. **Lan Ye:** Writing – original draft, Formal analysis, Data curation. **Carsten Buhmann:** Writing – review & editing, Data curation. **Fabian Maass:** Writing – review & editing, Data curation. **Christoph van Riesen:** Writing – review & editing, Data curation. **Christiane Weinrich:** Writing – review & editing, Data curation. **Florian Wegner:** Writing – review & editing. **Matthias Höllerhage:** Writing – review & editing. **Monika Pötter-Nerger:** Writing – review & editing, Data curation. **Martin Klietz:** Writing – review & editing, Writing – original draft, Conceptualization.

## Ethics declaration

Informed consent and patient details: Written informed consent to take part in the study and to publish the article has been obtained from all participants or their legal representatives. The privacy rights of participants have been observed.

Studies in Human: This study was performed in compliance with relevant laws, regulatory frameworks and guidelines where the research took place. This study was approved by the The Ethics Committee of Hannover Medical School. (Approval No. Hannover No. 866_Bo_K_2019)

Ethics statement: Ethical approval for this study was obtained from the Ethics Committee of Hannover Medical School (Hannover No. 866_Bo_K_2019). Patient Consent Statement Written informed consent was obtained for anonymised patient information to be published in this article. Funding information This research received no specific grant from any funding agency in the public, commercial, or not-for-profit sectors. Generative AI use Not appliable.

## Funding

This study received no external funding.

## Declaration of competing interest

The authors declare that they have no known competing financial interests or personal relationships that could have appeared to influence the work reported in this paper.
