# Supplementary material for: Impact of type 2 diabetes mellitus on acute levodopa responsiveness in Parkinson's disease: A Multicenter retrospective study
Source: Clin Park Relat Disord. 2026 Sep 15;15:100502. doi: 10.1016/j.prdoa.2026.100502 (PMC13627794; doi:10.1016/j.prdoa.2026.100502)
Supplement: Supplementary file 1 [file mmc2.docx]

Supplementary Table 1. Overview of the Antidiabetic Treatment

| Antidiabetic treatment | PD (n=76) | PD + PreDM (n=21) | PD + T2DM (n=39) |
| --- | --- | --- | --- |
| Insulin | 0 (0%) | 0 (0%) | 4 (10.3%) |
| SGLT2 inhibitors (Gliflozins) | 0 (0%) | 0 (0%) | 2 (5.1%) |
| Alpha-glucosidase inhibitors | 0 (0%) | 0 (0%) | 0 (0%) |
| Incretin-based therapies (GLP-1 receptor agonists, DPP-4 inhibitors) | 0 (0%) | 0 (0%) | 4 (10.3%) |
| Biguanides | 0 (0%) | 0 (0%) | 32 (82.1%) |
| Sulfonylureas | 0 (0%) | 0 (0%) | 1 (2.6 %) |
| No antidiabetics | 76 (100%) | 21 (100%) | 1 (2.6 %) |
| No information available | 0 (0%) | 0 (0%) | 2 (5.1%) |
